# Mesenchymal stem cell senescence as a potency brake: causes, consequences, and cures

**DOI:** 10.3389/fragi.2026.1929624

**Published:** 2026-08-17

**Authors:** Sen Zhou, Yu Xie, Yanjie Guo, Yufu Zhang, Yusi Liu

**Affiliations:** 1 Department of Immunology, Yan’an Medical College, Yan’an University, Yan’an, China; 2 Department of Hepatobiliary and Pancreatic Surgery, The Affiliated Hospital of Yan’an University, Yan’an, China

**Keywords:** cellular senescence, clinical translation, mesenchymal stem cells, rejuvenation strategies, therapeutic potency

## Abstract

Mesenchymal stem cells (MSCs) are widely investigated for regenerative medicine, tissue repair, immunomodulation, and selected cancer-related applications because of their multilineage differentiation potential, paracrine activity, immunoregulatory properties, and capacity to home to sites of injury. However, the therapeutic potential of MSCs depends not only on their tissue source or surface-marker expression but also on their functional state. MSC senescence is not a single phenotypic alteration; rather, it reflects the combined effects of persistent DNA damage response activation, disruption of mitochondrial and metabolic homeostasis, remodeling of epigenetic and secretory networks, and loss of proteostasis. Collectively, these changes drive MSCs toward reduced proliferative capacity, dysregulated paracrine signaling, impaired immunomodulatory activity, and diminished tissue-repair potential, thereby compromising cell-product potency and batch-to-batch consistency. This review summarizes the molecular mechanisms underlying MSC senescence, its source-specific functional consequences, and emerging rejuvenation strategies, with an emphasis on MSC quality control, potency assessment, and clinical translation.

## 1 Introduction

Mesenchymal stem cells (MSCs) are multipotent stromal/stem cells with multilineage differentiation potential, paracrine activity, immunomodulatory properties, and tissue-homing capacity (Zhou et al., 2021; Han et al., 2022; Wang Y. et al., 2022). They can be isolated from multiple tissues, including bone marrow, umbilical cord, adipose tissue, placenta, and dental pulp (Zhou et al., 2021; Li J. et al., 2023). Among these, bone marrow-derived MSCs, human umbilical cord-derived mesenchymal stem cells (hUC-MSCs), and adipose-derived MSCs are the most extensively studied and clinically applied. MSCs from different sources vary in harvesting procedures, expansion capacity, differentiation propensity, and intended clinical applications. However, their therapeutic effects are not determined solely by tissue origin. For MSCs, maintaining a favorable functional state is often more important than merely meeting basic phenotypic criteria (Li J. et al., 2023; Viswanathan and Galipeau, 2025).

Cellular senescence is a major contributor to functional decline in MSCs (Weng et al., 2022). Its triggers can be broadly classified into donor-related factors, manufacturing-related stressors, and microenvironmental challenges, including aging or disease, cumulative expansion and cryopreservation-related stress, and chronic inflammation, oxidative stress, or metabolic imbalance, respectively (Cottle et al., 2022; Wang et al., 2024). Although these triggers differ in origin, they converge on the three interrelated response axes considered in this review: persistent DNA damage response (DDR)-associated cell-cycle arrest; mitochondrial dysfunction coupled to reactive oxygen species (ROS) amplification and metabolic dysregulation; and epigenetic drift and proteostatic dysfunction accompanied by senescence-associated secretory phenotype (SASP) remodeling. Together, these processes can shift MSCs from a functionally competent state toward restricted proliferation, dysregulated secretion, and diminished reparative capacity (Weng et al., 2022; Wang et al., 2024; Liu et al., 2025). Accordingly, MSC senescence may be viewed as a potency-limiting factor relevant to cell-source selection, product release, and the reliability of clinical application (Weng et al., 2022).

This review examines MSC senescence from three perspectives: its molecular mechanisms, source-specific functional consequences, and rejuvenation strategies. The mechanistic section focuses on DDR-associated cell-cycle arrest, the mitochondrial dysfunction-reactive oxygen species (ROS)-metabolic dysregulation axis, and epigenetic drift, SASP remodeling, and proteostatic dysfunction, thereby outlining how MSCs transition from adaptive stress responses to stable senescence. The functional section compares the major processes impaired by senescence in bone marrow-derived MSCs, hUC-MSCs, and adipose-derived MSCs, highlighting both shared features and source-specific vulnerabilities. The intervention section summarizes approaches that optimize culture and manufacturing conditions, restore mitochondrial and metabolic homeostasis, and modulate epigenetic, autophagic, and paracrine networks, with the goal of supporting function-based quality evaluation and rejuvenation strategies (Weng et al., 2022; Sun et al., 2023; Lee et al., 2023; Wang et al., 2024; Liu et al., 2025).

## 2 Causes and molecular mechanisms of MSC senescence

**FIGURE 1:**
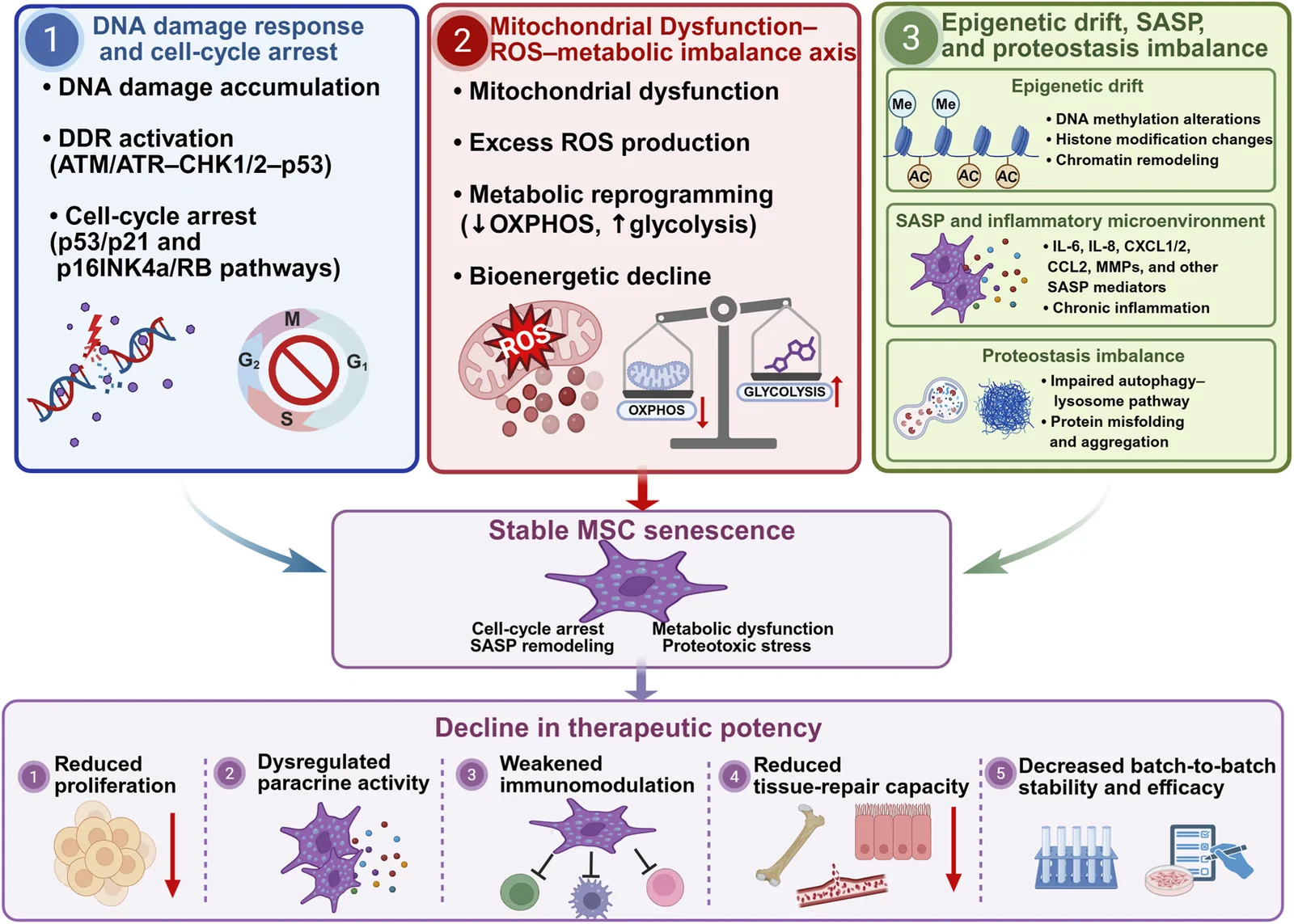
Major intracellular mechanisms driving mesenchymal stem cell senescence and therapeutic potency decline. Persistent DNA damage accumulation and DNA damage response (DDR) activation involving ATM/ATR–CHK1/2–p53 signaling promote p53/p21- and p16INK4a/RB-mediated cell-cycle arrest. In parallel, mitochondrial dysfunction increases reactive oxygen species (ROS) production, drives metabolic reprogramming characterized by reduced oxidative phosphorylation (OXPHOS) and increased glycolytic dependence, and contributes to bioenergetic decline. Epigenetic drift, senescence-associated secretory phenotype (SASP) remodeling, chronic inflammatory signaling, impaired autophagy–lysosome activity, and protein misfolding and aggregation further reinforce the senescent state. These interconnected mechanisms converge on stable MSC senescence, characterized by cell-cycle arrest, metabolic dysfunction, SASP remodeling, and proteotoxic stress, ultimately contributing to reduced proliferation, dysregulated paracrine activity, weakened immunomodulation, reduced tissue-repair capacity, and decreased batch-to-batch stability and therapeutic efficacy. Abbreviations: MSCs, mesenchymal stem cells; DDR, DNA damage response; ATM, ataxia-telangiectasia mutated; ATR, ATM and Rad3-related; CHK1/2, checkpoint kinase 1/2; ROS, reactive oxygen species; RB, retinoblastoma protein; OXPHOS, oxidative phosphorylation; SASP, senescence-associated secretory phenotype.

MSC senescence can be induced by donor-related factors, *in vitro* manufacturing stressors, and pathological microenvironmental conditions (Zhang et al., 2021). Although these triggers arise at different stages, they ultimately converge on shared intracellular processes, including increased genomic damage and repair burden, disruption of mitochondrial and metabolic homeostasis, and sustained remodeling of epigenetic and secretory networks (Weng et al., 2022; He et al., 2023; Wang et al., 2024; Ho et al., 2025). In other words, external triggers define the type of stress to which MSCs are exposed, whereas intracellular response mechanisms determine whether that stress is converted into a stable senescent state. As summarized in Figure 1, these interconnected pathways collectively drive MSCs from stress adaptation to stable senescence and ultimately contribute to reduced therapeutic potency. Accordingly, the mechanistic framework of MSC senescence is organized around the following three interrelated axes.

### 2.1 Persistent DNA damage response signaling and cell-cycle arrest in replicative senescence

Persistent activation of the DNA damage response (DDR) is a key mechanism underlying replicative senescence in MSCs. With increasing donor age or cumulative *in vitro* passaging, telomere shortening, replication stress, oxidative base damage, and DNA double-strand breaks gradually accumulate. Cells initiate damage recognition, cell-cycle arrest, and repair programs through signaling mediated by ataxia-telangiectasia mutated (ATM), ATM and Rad3-related (ATR), phosphorylated histone H2AX (γH2AX), and checkpoint kinase 1/2 (CHK1/CHK2). However, when DNA damage exceeds repair capacity or repair signaling remains persistent, the DDR shifts from a transient protective response to a mechanism that sustains senescence. At this stage, the p53/p21 pathway inhibits cyclin-dependent kinase (CDK) activity and blocks progression from G1 to S phase, whereas the p16INK4a/retinoblastoma protein (RB) pathway suppresses E2F transcription factor-dependent expression of proliferation-related genes by maintaining RB in a hypophosphorylated state. Together, these pathways drive cells from reversible stress-induced arrest to stable cell-cycle arrest (Xia et al., 2020; Wang S. et al., 2021; Kim and Kim, 2024; Taherian Fard et al., 2024).

Cell-cycle arrest initially manifests as reduced proliferation and colony-forming ability but can subsequently compromise differentiation, migration, and paracrine function. Sustained activation of the p53/p21 and p16INK4a/RB pathways reduces expansion efficiency, making it difficult for MSCs to achieve the cell doses required for experimental or clinical use within a reasonable time frame. In parallel, DDR-associated transcriptional changes may affect lineage regulators such as runt-related transcription factor 2 (RUNX2), SRY-box transcription factor 9 (SOX9), and peroxisome proliferator-activated receptor gamma (PPARγ), as well as migration- and paracrine-related molecules including C-X-C chemokine receptor type 4 (CXCR4), matrix metalloproteinases (MMPs), vascular endothelial growth factor (VEGF), and hepatocyte growth factor (HGF). Consequently, even MSCs that retain canonical surface markers may exhibit impaired osteogenic or chondrogenic differentiation, reduced homing to injured tissue, and insufficient secretion of pro-reparative factors (Xia et al., 2020; Wang S. et al., 2021; Weng et al., 2022; Kim and Kim, 2024; Wang et al., 2024). More importantly, persistent DDR can promote SASP formation through stress pathways such as p38 mitogen-activated protein kinase (p38 MAPK) and nuclear factor kappa B (NF-κB), gradually shifting the secretory profile from a repair-supportive state to a pro-inflammatory state associated with aberrant matrix remodeling (Chou et al., 2022).

Thus, in MSC quality assessment, DNA damage may be considered not only a senescence marker but also a mechanistic indicator linking donor age and *in vitro* expansion stress to potential losses in therapeutic potency. Assessment based only on cell viability or surface-marker expression may not fully capture the DDR burden. Evaluation of replicatively senescent MSCs can therefore be strengthened by integrating passage number, γH2AX, p53/p21, p16INK4a/RB, telomere status, colony-forming ability, and differentiation and migration functions (Zhai et al., 2021; Yang et al., 2023). Persistent DDR is therefore not an isolated pathway; mitochondrial dysfunction and excessive ROS production can both initiate and reinforce DNA damage, creating the metabolic feedback loop discussed in the following section.

### 2.2 Mitochondrial dysfunction and ROS amplification drive oxidative stress and metabolic dysregulation

Mitochondrial dysfunction is repeatedly observed across MSC senescence models and is closely linked to functional decline. In addition to generating adenosine triphosphate (ATP), mitochondria regulate redox balance, calcium homeostasis, apoptosis, innate immune activation, and stress adaptation. With increasing passage number, or under metabolic stressors such as inflammatory stimulation, hypoxia/reoxygenation, high glucose, and high lipid exposure, MSCs may exhibit reduced mitochondrial membrane potential, decreased electron-transport-chain efficiency, diminished ATP production, mitochondrial swelling or fragmentation, mitochondrial DNA damage, and impaired mitochondrial quality control. These alterations increase electron leakage, making mitochondria a major source of ROS accumulation (Yan et al., 2021; Shen et al., 2024; Tan et al., 2025).

ROS play a dual role in MSC senescence. At physiological levels, ROS act as signaling molecules that support differentiation, migration, and stress adaptation. In contrast, persistent ROS excess oxidizes DNA, proteins, and lipids while further damaging mitochondria, forming a self-amplifying loop of mitochondrial injury, ROS accumulation, and additional mitochondrial damage. This feed-forward loop enhances DDR signaling and reinforces activation of the p53/p21 and p16INK4a/RB pathways (Liu et al., 2023; Wang Y. et al., 2023; Shen et al., 2024). It can also promote an inflammatory secretory phenotype through pathways involving NF-κB, the NLR family pyrin domain containing 3 (NLRP3) inflammasome, and p38 MAPK. Moreover, when damaged mitochondria are not efficiently eliminated through PTEN-induced kinase 1 (PINK1)/Parkin-mediated mitophagy or other quality-control mechanisms, they may continuously release intracellular danger signals that further stabilize the senescent state (Lee et al., 2020; Fan et al., 2022; Li W. et al., 2024).

Mitochondrial dysfunction is also tightly linked to metabolic regulatory networks involving nicotinamide adenine dinucleotide (NAD^+^)/sirtuin signaling, AMP-activated protein kinase (AMPK), and mechanistic target of rapamycin (mTOR). Declining NAD^+^ levels reduce the activity of deacetylases such as sirtuin 1 (SIRT1) and sirtuin 3 (SIRT3), thereby affecting DNA repair, mitochondrial biogenesis, antioxidant defense, and inflammatory regulation. Insufficient AMPK activity or excessive mTOR signaling can impair energy sensing, autophagic clearance, and proteostasis maintenance. Because MSC migration, paracrine activity, and immunomodulation depend on stable energy metabolism and redox balance, mitochondrial dysfunction ultimately manifests as reduced homing capacity, dysregulated paracrine profiles, weakened anti-inflammatory activity, and inconsistent tissue-repair outcomes. Therefore, MSC rejuvenation studies should evaluate mitochondrial membrane potential, ATP, ROS/mitochondrial ROS, NAD^+^ levels, sirtuin activity, and mitophagy markers alongside functional assays; ROS reduction alone should not be considered a sufficient endpoint (Yan et al., 2021; Wen et al., 2025). However, restoration of redox and metabolic homeostasis may remain incomplete when stress-induced transcriptional programs become epigenetically stabilized, providing a mechanistic basis for senescence-associated memory and persistent SASP remodeling.

### 2.3 Epigenetic drift and proteostatic dysfunction sustain SASP remodeling and senescence-associated memory

In addition to genomic damage and metabolic dysregulation, epigenetic drift helps explain why senescent MSCs are difficult to fully restore. Long-term passaging, inflammatory stimulation, oxidative stress, and metabolite changes can alter DNA methylation, histone acetylation and methylation, chromatin accessibility, and non-coding RNA expression, thereby reshaping transcriptional programs governing proliferation, differentiation, antioxidant responses, inflammation, and paracrine activity (Potter et al., 2021; Shi et al., 2021; Sun et al., 2023; Chen et al., 2024; Jin et al., 2024; Ho et al., 2025). Unlike transient signaling events, epigenetic alterations can persist and translate short-lived external stimuli into relatively stable gene-expression programs. Thus, MSCs may retain a senescence-associated memory even after removal from the original stress environment (Potter et al., 2021; Sun et al., 2023; Chen et al., 2024; Jin et al., 2024; Ho et al., 2025).

SASP represents the extracellular functional manifestation of this persistent state. Senescent MSCs may secrete higher levels of inflammatory mediators, chemokines, matrix-remodeling enzymes, and stress-associated molecules, while the secretion of pro-reparative, pro-angiogenic, and immunomodulatory factors may decline (Zheng et al., 2023; Boulestreau et al., 2024). On the one hand, SASP influences neighboring immune cells, endothelial cells, fibroblasts, and tissue parenchymal cells through paracrine mechanisms, thereby reshaping the local inflammatory and reparative microenvironment. On the other hand, SASP can act in an autocrine manner to sustain senescence through pathways involving interleukin-6 (IL-6)/signal transducer and activator of transcription 3 (STAT3), NF-κB, transforming growth factor-beta (TGF-β), and p38 MAPK. Thus, senescent MSCs are not merely functionally compromised; they may actively remodel the microenvironment of target tissue, shifting regeneration and repair toward chronic inflammation, fibrosis, or matrix degradation (Chou et al., 2022; Boulestreau et al., 2024; Liu et al., 2024).

Proteostasis regulatory systems, including autophagy, the proteasome, and endoplasmic reticulum stress responses, also contribute to the maintenance of senescence (Llewellyn et al., 2023; Zheng et al., 2023). Insufficient autophagy decreases the clearance of damaged mitochondria and abnormal proteins, proteasome dysfunction aggravates the accumulation of misfolded proteins, and persistent endoplasmic reticulum stress can further activate inflammatory and apoptosis-related signaling. Therefore, epigenetic drift, SASP remodeling, and proteostatic dysfunction are not isolated phenomena; together, they constitute a network that sustains MSC senescence. This framework explains why reducing a single senescence marker may not restore therapeutic function and suggests that rejuvenation strategies should simultaneously address intracellular damage clearance, transcriptional-state remodeling, and restoration of paracrine function (Shi et al., 2021; Llewellyn et al., 2023; Boulestreau et al., 2024). Although these mechanisms are broadly shared across MSC populations, their relative contribution and functional consequences vary according to tissue source, donor background, and manufacturing history.

## 3 Source-specific consequences of MSC senescence for therapeutic potency and clinical translation

**FIGURE 2:**
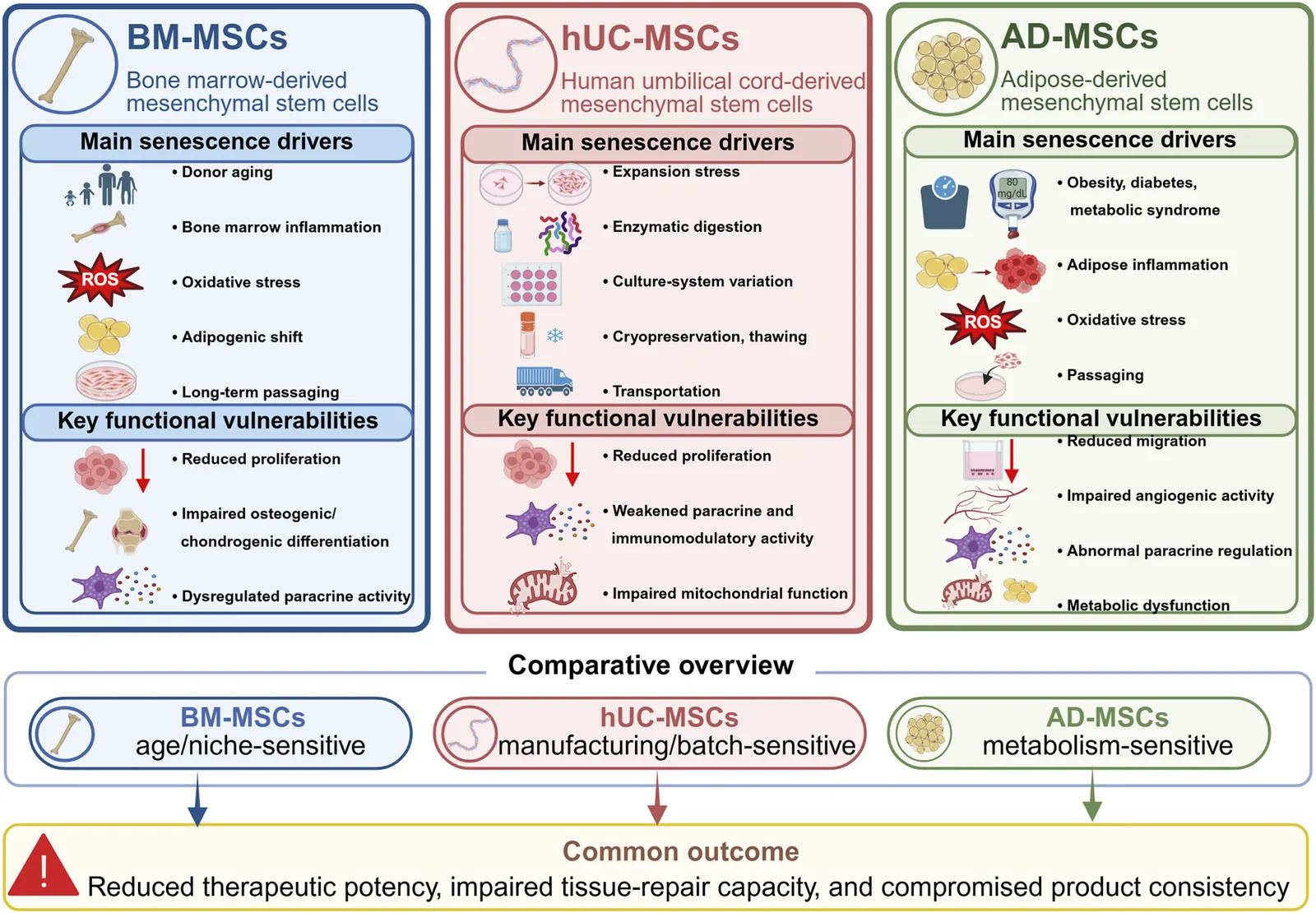
Source-specific functional consequences of mesenchymal stem cell senescence. Bone marrow-derived MSCs (BM-MSCs) are particularly affected by donor aging, bone marrow inflammation, oxidative stress, adipogenic shift, and long-term passaging, which are commonly associated with reduced proliferation, impaired osteogenic/chondrogenic differentiation, and dysregulated paracrine activity. Human umbilical cord-derived MSCs (hUC-MSCs) are more strongly influenced by manufacturing-related stressors, including expansion stress, enzymatic digestion, culture-system variation, cryopreservation, thawing, and transportation, which may compromise proliferation, paracrine and immunomodulatory activity, and mitochondrial function. Adipose-derived MSCs (AD-MSCs) are particularly affected by obesity, diabetes, metabolic syndrome, adipose inflammation, oxidative stress, and passaging, resulting in reduced migration, impaired angiogenic activity, abnormal paracrine regulation, and metabolic dysfunction. Thus, BM-MSC senescence is relatively age- and niche-sensitive, hUC-MSC senescence is predominantly manufacturing- and batch-sensitive, and AD-MSC senescence is closely associated with metabolic stress. Despite these source-specific differences, MSC senescence converges on reduced therapeutic potency, impaired tissue-repair capacity, and compromised product consistency. Abbreviations: MSCs, mesenchymal stem cells; BM-MSCs, bone marrow-derived mesenchymal stem cells; hUC-MSCs, human umbilical cord-derived mesenchymal stem cells; AD-MSCs, adipose-derived mesenchymal stem cells.

The therapeutic value of MSCs mainly depends on their expansion capacity, migratory ability, paracrine activity, immunomodulatory function, and tissue-repair potential (Han et al., 2025). MSCs from different sources vary in harvesting procedures, baseline functional states, and intended disease indications. Consequently, the functions that are most susceptible to senescence and the resulting impact on therapeutic potency are not identical across cell sources (Turlo et al., 2023; Hori et al., 2024). Differences in tissue microenvironment, manufacturing workflow, and clinical use also shape the dominant senescence mechanisms and functional vulnerabilities of bone marrow-derived MSCs, hUC-MSCs, and adipose-derived MSCs (Hoseini and Montazeri, 2025). Figure 2 provides a comparative overview of source-specific senescence-related stressors and major functional vulnerabilities. Despite these differences, MSC senescence ultimately converges on reduced therapeutic potency and diminished tissue-repair capacity.

### 3.1 Bone marrow-derived MSCs: age- and niche-related senescence limits bone and hematopoietic repair

Bone marrow-derived mesenchymal stem cells (BM-MSCs) are typically obtained from adult bone marrow, and their functional state is strongly influenced by donor age, underlying disease, and alterations in the bone marrow microenvironment. With aging, chronic inflammation, oxidative stress, adipogenic bias, and metabolic dysregulation progressively intensify within the bone marrow, such that BM-MSCs may already be pre-senescent or functionally compromised before isolation (Deng et al., 2021; Peng et al., 2022; Selle et al., 2022). Although *in vitro* expansion increases cell number, it also adds replicative stress, leading to telomere shortening, DDR activation, mitochondrial dysfunction, and activation of the p53/p21 and p16INK4a/RB pathways. These changes ultimately result in reduced proliferation, diminished colony-forming ability, and impaired osteogenic and chondrogenic differentiation potential (Deng et al., 2021; Li et al., 2021; Selle et al., 2022).

BM-MSCs are closely associated with bone and cartilage tissues and with the hematopoietic niche. They are therefore widely studied in bone defects, osteoporosis, osteoarthritis, cartilage injury, and disorders involving damage to the hematopoietic microenvironment. Their therapeutic activity depends not only on direct osteogenic or chondrogenic differentiation but also on secretion of pro-reparative mediators, regulation of local inflammation, support of extracellular-matrix formation, and maintenance of bone marrow homeostasis (Massaro et al., 2023; Mitchell et al., 2023). Because BM-MSCs are tightly coupled to the bone marrow niche, their therapeutic function is particularly sensitive to donor age, marrow inflammation, and *in vitro* expansion history (Selle et al., 2022; Massaro et al., 2023; Mitchell et al., 2023).

During BM-MSC senescence, expansion efficiency and differentiation capacity are often among the earliest functions to deteriorate. Cells from older donors or high-passage cultures may fail to yield sufficient cell numbers within a practical time frame, and their osteogenic and chondrogenic potential may decline, directly compromising bone and cartilage regeneration (Deng et al., 2021; Li et al., 2021; Peng et al., 2022). Meanwhile, the paracrine profile of senescent BM-MSCs may shift from a pro-reparative pattern toward pro-inflammatory, matrix-degradative, or adipogenic programs, undermining the consistency of their effects in degenerative bone disease and bone marrow microenvironment injury (Peng et al., 2022; Massaro et al., 2023; Mitchell et al., 2023). Accordingly, BM-MSC quality assessment should extend beyond surface-marker expression to include donor age, passage number, colony-forming ability, osteogenic and chondrogenic potential, mitochondrial function, and the inflammatory status of the bone marrow niche (Li et al., 2021; Selle et al., 2022; Hellmich et al., 2023). In contrast to the strong donor-age and niche dependence of BM-MSCs, the major vulnerabilities of hUC-MSCs arise primarily during isolation, expansion, banking, thawing, and distribution.

### 3.2 Human umbilical cord-derived MSCs: Manufacturing stress and batch consistency affect allogeneic therapeutic potency

Human umbilical cord-derived mesenchymal stem/stromal cells (hUC-MSCs) are most commonly isolated from umbilical cord tissue, particularly Wharton’s jelly, and should be distinguished from human umbilical cord blood-derived mesenchymal stromal cells (hUCB-MSCs). Compared with adult tissue-derived MSCs, hUC-MSCs generally exhibit a younger baseline state, greater proliferative capacity, and lower immunogenicity, making them attractive for allogeneic cell therapy and large-scale manufacturing (Mebarki et al., 2021; Wang X. et al., 2023; Chu et al., 2024). However, hUC-MSCs are not intrinsically resistant to senescence. Prolonged expansion, enzymatic digestion, variation in seeding density, differences in culture systems, cryopreservation and thawing, and transportation can collectively impose manufacturing stress, resulting in slower proliferation, altered morphology, reduced mitochondrial function, and impaired paracrine activity (Nguyen et al., 2021; Wang X. et al., 2023; Chu et al., 2024).

hUC-MSCs are widely investigated in immune and inflammatory diseases, lung injury, liver injury, neurological injury, tissue repair, and selected cancer-related applications. Relative to BM-MSCs, hUC-MSCs are often valued in immune-inflammatory and tissue-protective settings for their paracrine and immunomodulatory effects (Nguyen et al., 2021; Kim et al., 2023; Chu et al., 2024). Through cytokines, chemokines, extracellular vesicles, and other mediators, they can influence immune cells, endothelial cells, and injured tissue cells, thereby exerting anti-inflammatory, anti-apoptotic, pro-angiogenic, and tissue-protective effects. Accordingly, the key quality question for hUC-MSCs is not simply whether they can be expanded, but whether their secretory and immunomodulatory functions remain stable after expansion, cryopreservation, and thawing (Nguyen et al., 2021; Mebarki et al., 2025).

Because hUC-MSCs are obtained from perinatal donors, they are not subject to the same degree of chronological donor aging as adult tissue-derived MSCs; however, their functional consistency may still be influenced by donor variability, isolation site, expansion conditions, cryopreservation and thawing, and transportation procedures (Cottle et al., 2022; Wang X. et al., 2023; Chu et al., 2024; Mebarki et al., 2025). For products manufactured at scale, focusing only on cell number, viability, and surface-marker expression while overlooking post-thaw or high-passage changes in secretory function may lead to batch-to-batch variation in therapeutic potency (Nguyen et al., 2021; Cottle et al., 2022; Mebarki et al., 2025). When senescent hUC-MSCs exhibit reduced anti-inflammatory factor secretion, increased pro-inflammatory signaling, or lower levels of pro-angiogenic and tissue-protective mediators such as VEGF and HGF, their immunomodulatory effects in inflammatory disease and their anti-apoptotic, pro-angiogenic, or anti-fibrotic activity in tissue repair may be compromised. Therefore, despite their relatively youthful origin, hUC-MSCs should be assessed with attention to manufacturing history, pre-administration handling, paracrine function, immunosuppressive capacity, and batch-to-batch consistency.

An important distinction should also be made between MSCs derived from umbilical cord tissue and those recovered from umbilical cord blood. MSCs have been isolated from cryopreserved umbilical cord blood units, although the cited study reported a lower isolation rate than that obtained from non-cryopreserved cord blood, and current evidence does not establish a direct relationship between cryostorage duration and cellular senescence (Fujii et al., 2017). Consequently, cryostorage duration should be documented as a manufacturing variable rather than treated as an established independent cause of senescence. Its potential effects should be evaluated together with pre-freeze processing, cryoprotectant exposure, cooling and thawing protocols, temperature excursions, and post-thaw expansion. Future studies should compare defined storage durations under otherwise standardized conditions and assess senescence markers alongside proliferative capacity, mitochondrial function, paracrine activity, and immunomodulatory potency. Whereas hUC-MSC quality is strongly shaped by manufacturing-related variables, AD-MSC potency is particularly influenced by the metabolic and inflammatory status of the donor.

### 3.3 Adipose-derived MSCs: metabolic-inflammatory microenvironments compromise soft-tissue repair and paracrine function

Adipose-derived mesenchymal stem cells (AD-MSCs) are isolated from adipose tissue, and their susceptibility to senescence is closely linked to donor metabolic status. Adipose tissue is not only an energy-storage organ but also an active endocrine and immunoregulatory compartment. In obesity, diabetes, metabolic syndrome, or chronic inflammatory states, inflammatory mediators, free fatty acids, and oxidative stress are elevated in adipose tissue; thus, AD-MSCs may already be conditioned by an adverse microenvironment before isolation. Subsequent *in vitro* passaging, cryopreservation and thawing, and oxidative stress exposure can further exacerbate replicative senescence and functional decline (Ren et al., 2021; Grun et al., 2023; Li C. et al., 2024).

AD-MSCs are relatively accessible and typically provide a high initial cell yield. They are widely investigated for soft-tissue repair, wound healing, angiogenesis, immunomodulation, metabolic diseases, and aesthetic regenerative medicine. Compared with BM-MSCs, AD-MSCs offer practical advantages in tissue accessibility and initial cell yield while retaining substantial paracrine and microenvironment-modulating capacity. Their therapeutic value is largely attributed to promotion of local angiogenesis, regulation of inflammatory responses, modulation of extracellular-matrix remodeling, and support of tissue repair (Schneider et al., 2023).

Senescence in AD-MSCs can reduce migratory ability, pro-angiogenic capacity, and paracrine regulatory activity (Grun et al., 2023; Li R. et al., 2023; Li C. et al., 2024). In wound healing and soft-tissue repair, inadequate recruitment to injured sites, weakened angiogenic signaling, or impaired matrix-regulatory capacity can compromise both the rate and quality of repair. The metabolic background of the donor is particularly important in autologous AD-MSC applications. Although autologous cells offer an immunologic matching advantage, AD-MSCs from individuals with obesity, diabetes, or metabolic syndrome may already display inflammation- and metabolic stress-associated injury, with suboptimal anti-inflammatory, pro-reparative, and paracrine activity (Ren et al., 2021; Grun et al., 2023; Foti et al., 2024; Li C. et al., 2024). Accordingly, AD-MSC assessment should extend beyond cell yield to integrate donor metabolic status, adipose tissue inflammation, mitochondrial function, migratory capacity, and paracrine assay results (Ren et al., 2021; Grun et al., 2023; Foti et al., 2024; Li C. et al., 2024).

## 4 Cures: rejuvenation strategies for senescent MSCs

The goal of MSC rejuvenation is not simply to increase proliferative rate but to restore functions directly relevant to therapeutic action while preserving cell identity, genetic stability, and safety. Current approaches primarily aim to reduce *in vitro* manufacturing stress, restore mitochondrial and metabolic homeostasis, and remodel epigenetic, autophagic, and paracrine networks (Lee et al., 2023). Evaluation of these interventions should consider both changes in senescence markers and recovery of therapy-relevant functions; improvement in a single molecular readout should not be equated directly with enhanced clinical potency (Maillot et al., 2021).

### 4.1 Optimizing culture and manufacturing systems to reduce *in vitro* expansion stress

*In vitro* expansion is indispensable for most clinical MSC applications but is also a major driver of cellular senescence. BM-MSCs, hUC-MSCs, and AD-MSCs all require *ex vivo* culture to generate sufficient cell numbers for experimental or therapeutic use. However, prolonged passaging increases replicative and oxidative stress, progressively leading to reduced proliferative capacity, enlarged morphology, cell-cycle arrest, and functional decline. Therefore, strategies to preserve MSC function should be implemented as early as possible within the manufacturing workflow, minimizing expansion stress rather than attempting to rescue cells after overt senescence has developed (Maillot et al., 2021; Egger et al., 2022).

Culture and manufacturing optimization may include hypoxic culture, three-dimensional culture, optimized seeding density, control of passage number, refinement of medium composition, and the establishment of serum-free or animal component-free culture systems. Hypoxic culture can reduce the oxidative burden associated with conventional high-oxygen culture and better approximate the physiologic oxygen niche *in vivo*. Three-dimensional culture can enhance cell-cell interactions and improve selected features of paracrine signaling and extracellular vesicle (EV) release (Regmi et al., 2021). Careful control of seeding density, confluence, and passage timing helps limit phenotypic and functional variation caused by fluctuating culture conditions (Egger et al., 2022; López-Fernández et al., 2024). Serum-free or animal component-free systems can improve the safety and batch-to-batch consistency of clinical manufacturing (Wu et al., 2023; López-Fernández et al., 2024). For products such as hUC-MSCs that require large-scale production, culture-system stability is a major determinant of batch consistency (Aabling et al., 2023; López-Fernández et al., 2024). For BM-MSCs and AD-MSCs, controlling passage number and limiting expansion stress likewise help preserve reparative potential.

Cryopreservation, thawing, and transportation should also be incorporated into quality-control strategies designed to preserve a youthful MSC state (Aabling et al., 2023). Some MSCs may appear functionally competent before cryopreservation but display reduced mitochondrial membrane potential, increased ROS, delayed recovery of adhesion, or impaired paracrine capacity after thawing. If clinical evaluation relies only on post-thaw viability without assessing migration, secretion, and immunomodulatory function, the potency of the product may be overestimated (François et al., 2012; Nguyen et al., 2021; Cottle et al., 2022; Aabling et al., 2023). Thus, optimization of culture and manufacturing is not merely a process-development issue; it is central to maintaining the functional fitness of MSCs and establishing a meaningful potency-assessment framework. Manufacturing optimization can prevent additional stress and functional deterioration; however, MSCs with established mitochondrial and metabolic dysfunction may require direct interventions targeting redox balance and organelle quality control.

Because many MSC products are administered soon after thawing, post-thaw potency assessment should reproduce the clinically relevant handling window. A retained aliquot from each batch may be evaluated immediately after thawing and, where applicable, at the end of the validated post-thaw holding period. Rapid release-related measures may include viable-cell recovery, early apoptosis, adhesion recovery, mitochondrial membrane potential, ATP content, and validated secreted or cell-surface surrogate markers. Mechanism-linked assays should be selected according to the intended indication: suppression of activated T-cell or peripheral blood mononuclear cell (PBMC) proliferation, interferon-γ (IFN-γ)-induced indoleamine 2,3-dioxygenase (IDO) activity or kynurenine production, and prostaglandin E2 (PGE2) secretion for immunomodulatory applications; endothelial-cell migration or tube formation and VEGF/HGF secretion for angiogenic or tissue-repair applications; and chemotaxis or adhesion assays when tissue homing is essential (Robb et al., 2022; Sagaradze et al., 2023; Davies et al., 2025; Viswanathan and Galipeau, 2025). Freshly thawed MSCs can show transient impairment of IFN-γ licensing and T-cell suppression that recovers after culture, illustrating why post-thaw timing must be standardized (François et al., 2012). Because many functional assays require prolonged incubation, they are better suited to batch characterization, comparability, or process validation using retained aliquots than to same-day real-time release. A matrix combining rapid release-related measurements with indication- and mechanism-linked potency assays is therefore preferable to any single test.

### 4.2 Improving mitochondrial function, redox balance, and metabolic homeostasis

Interventions targeting mitochondrial dysfunction and ROS accumulation can be grouped into three categories according to their principal mode of action. The first includes small-molecule antioxidant and anti-stress interventions that reduce excessive ROS and alleviate mitochondrial injury. The second includes NAD^+^ supplementation and sirtuin activation to support energy metabolism, deacetylation-dependent regulation, and DNA repair. The third includes mitochondria-targeted protection and enhancement of quality-control processes to remove or stabilize damaged mitochondria. These approaches are interrelated but mechanistically distinct and should be evaluated as complementary rejuvenation strategies (Wang J. et al., 2021; Zhong et al., 2021; Wang H. et al., 2022; Luo et al., 2024; Peng et al., 2024; Vikraman et al., 2026).

Among small-molecule antioxidant and anti-stress approaches, melatonin, resveratrol, vitamin C, and coenzyme Q10 are commonly investigated (Thaler et al., 2022; Yi et al., 2022; Li et al., 2025; Vikraman et al., 2026). Beyond scavenging free radicals and exerting anti-inflammatory effects, melatonin can promote mitophagy through pathways involving heat shock protein family A member 1-like (HSPA1L)/Parkin and improve mitochondrial quality control under oxidative stress (Lee et al., 2020; Vikraman et al., 2026). Resveratrol can alleviate inflammatory and oxidative injury by activating SIRT1/AMPK, inhibiting NF-κB, and modulating mTOR-related signaling (Li et al., 2025). Vitamin C reduces ROS and may also influence DNA methylation and gene expression by affecting ten-eleven translocation (TET) dioxygenases (Thaler et al., 2022). Coenzyme Q10 supports electron transport and mitochondrial membrane stability, thereby contributing to energy generation and redox balance (Vikraman et al., 2026). These interventions are commonly associated with reduced ROS, improved mitochondrial membrane potential, decreased apoptosis, and partial restoration of proliferative, migratory, or osteogenic differentiation capacity.

NAD^+^ supplementation and sirtuin-related strategies have become a major focus of MSC rejuvenation research. Nicotinamide mononucleotide (NMN), nicotinamide riboside (NR), nicotinamide, and exogenous NAD^+^ can raise intracellular NAD^+^ levels and influence SIRT1/SIRT3-mediated mitochondrial homeostasis, DNA repair, antioxidant responses, and inflammatory regulation. Available studies indicate that NMN can improve mitochondrial function and alleviate senescence in late-passage MSCs through the NAD^+^/SIRT3 pathway (Wang H. et al., 2022), whereas exogenous NAD^+^ can reduce ROS and senescence-associated phenotypes through SIRT1 signaling in D-galactose-induced BM-MSC senescence models (Wang J. et al., 2021). These findings provide mechanistic support for the premise that restoration of mitochondrial function can improve therapy-relevant MSC properties. Nevertheless, the evidence remains largely limited to *in vitro* and preclinical animal models, and reproducibility across MSC sources, manufacturing systems, and disease indications requires further investigation.

Mitochondria-targeted protection and enhancement of quality-control pathways focus more directly on damaged organelles. Mitochondria-targeted antioxidants such as mitoquinone (MitoQ) can reduce ROS and mitochondrial injury in oxidative stress-mediated BM-MSC senescence models (Zhong et al., 2021). Enhancing mitophagy or autophagy-lysosome function can likewise restore cellular homeostasis by clearing damaged organelles. Recent studies of transcription factor EB (TFEB)-mediated autophagy enhancement, small-molecule autophagy activators, and young-cell-derived small extracellular vesicles that regulate dynamin-related protein 1 (DRP1)-associated mitochondrial dynamics suggest that mitochondrial quality control has evolved from an observational marker into an actionable rejuvenation target (Luo et al., 2024; Peng et al., 2024). However, indiscriminate suppression of ROS may be counterproductive because basal ROS signaling contributes to normal MSC differentiation and stress adaptation. Therefore, evaluation of these strategies should include ROS, mitochondrial membrane potential, ATP, mitophagy, migration, paracrine activity, and immunomodulatory function, with functional recovery serving as a key endpoint.

### 4.3 Remodeling epigenetic, proteostatic, and paracrine networks

Epigenetic regulation, autophagy/proteostasis, and paracrine function are not independent intervention categories; rather, they correspond to the transcriptional program, damage-clearance capacity, and therapeutic output of senescent MSCs, respectively. Epigenetic drift can convert transient stress caused by prolonged passaging or persistent microenvironmental stimulation into relatively stable changes in gene expression and therefore represents an important basis for senescence-associated memory. Vitamin C, regulation of histone modifications, non-coding RNA interventions, and partial reprogramming have all been explored as approaches to improve the function of senescent MSCs. Vitamin C may influence DNA methylation by modulating redox status and the activity of TET dioxygenases. MicroRNAs (miRNAs), long non-coding RNAs (lncRNAs), and circular RNAs (circRNAs) can regulate proliferation, differentiation, and SASP by targeting pathways such as p53/p21, p16INK4a/RB, SIRT1, Wnt/β-catenin, phosphoinositide 3-kinase/protein kinase B (PI3K/AKT), and NF-κB (Potter et al., 2021; Cai et al., 2021; Shi et al., 2021; Chen et al., 2023). Short-term, controllable partial reprogramming induced by OCT4, SOX2, KLF4, and c-MYC (OSKM) has also been explored to reduce epigenetic age and improve the state of late-passage MSCs (Ivanova et al., 2024). However, these approaches may alter cell identity or introduce genetic and epigenetic instability and tumor-promoting risk; thus, they should be advanced only in rigorously controlled experimental systems.

As the epigenetic program is remodeled, intracellular clearance systems determine whether damaged mitochondria, abnormal proteins, and metabolic waste are eliminated efficiently. Enhancement of autophagy or mitophagy can reduce ROS-generating sources and inflammatory signaling and is closely interconnected with NAD^+^/sirtuin, AMPK/mTOR, and PINK1/Parkin pathways. mTOR inhibition, AMPK activation, sirtuin modulation, TFEB nuclear translocation, and enhancement of PINK1/Parkin signaling may improve proteostasis and organelle quality control in senescent MSCs (Lee et al., 2020; Wang J. et al., 2021; Fan et al., 2022; Wang H. et al., 2022; Hwang et al., 2024; Li W. et al., 2024; Luo et al., 2024). For example, CXM102 promotes TFEB nuclear translocation and autophagy, enhances the osteogenic tendency of senescent BM-MSCs, and has shown potential to improve bone mass and health-related outcomes in middle-aged mice (Luo et al., 2024).

Paracrine function is a major therapeutic output through which intracellular rejuvenation is translated into tissue-level effects (Hwang et al., 2024; Chen et al., 2025). Rejuvenation strategies should therefore assess not only whether markers such as senescence-associated β-galactosidase (SA-β-gal), p16/p21, or ROS decline, but also whether the quality of cytokines, chemokines, and extracellular vesicles (EVs) improves. Common enhancement strategies include genetic engineering, pharmacological pretreatment, and optimization of culture conditions. Genetic engineering can target VEGF, HGF, CXCR4, SIRT1, hypoxia-inducible factor-1α (HIF-1α), and related pathways to enhance pro-angiogenic, injury-homing, antioxidant, or tissue-protective functions of MSCs (Christoffers et al., 2024). Pharmacological pretreatment with melatonin, NAD^+^ precursors, antioxidants, anti-inflammatory agents, or hypoxic conditioning may improve cellular stress resistance. Culture conditions such as hypoxia, three-dimensional culture, and inflammatory-factor priming can likewise reshape the secretory profile and EV cargo (Regmi et al., 2021; Chu et al., 2023; Chen et al., 2025). MSC-derived EVs represent a cell-free therapeutic modality that may reduce some risks associated with live-cell transplantation and can influence senescent cells and injured tissue through the transfer of miRNAs, proteins, and metabolic regulatory signals. Available evidence suggests that small EVs derived from young cells can improve multipotency, immunomodulatory capacity, and mitochondrial dynamics in replicatively senescent BM-MSCs (Peng et al., 2024). Future rejuvenation strategies should therefore prioritize paracrine quality, EV function, and disease-relevant potency assays rather than phenotypic rejuvenation alone.

**FIGURE 3:**
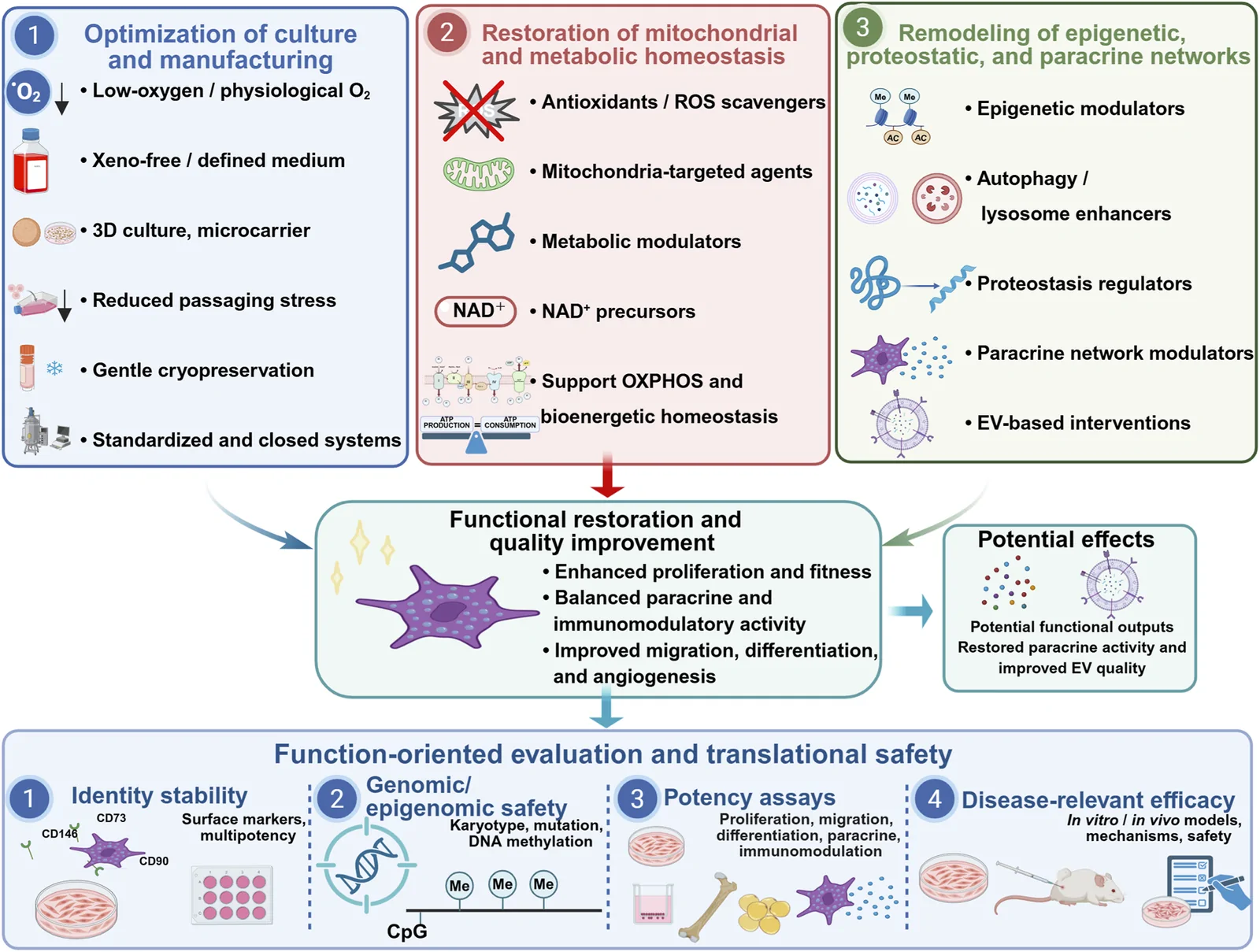
Rejuvenation strategies, potential functional outputs, and function-oriented evaluation for senescent mesenchymal stem cells. MSC rejuvenation can be pursued through optimization of culture and manufacturing conditions, restoration of mitochondrial and metabolic homeostasis, and remodeling of epigenetic, proteostatic, and paracrine networks. Culture- and manufacturing-related strategies include low-oxygen or physiologic oxygen culture, xeno-free or defined media, three-dimensional culture, microcarrier-based expansion, reduced passaging stress, gentle cryopreservation, and standardized closed systems. Mitochondrial and metabolic interventions include antioxidants or reactive oxygen species (ROS) scavengers, mitochondria-targeted agents, metabolic modulators, nicotinamide adenine dinucleotide (NAD^+^) precursors, and strategies supporting oxidative phosphorylation (OXPHOS) and bioenergetic homeostasis. Epigenetic-proteostasis-paracrine interventions include epigenetic modulators, autophagy/lysosome enhancers, proteostasis regulators, paracrine network modulators, and extracellular vesicle (EV)-based interventions. These approaches may promote functional restoration and quality improvement, including enhanced proliferative fitness, balanced paracrine and immunomodulatory activity, and improved migration, differentiation, and angiogenesis, while potentially restoring paracrine activity and improving EV quality. Successful rejuvenation should be evaluated using a function-oriented and safety-anchored framework incorporating identity stability, genomic/epigenomic safety, disease-relevant potency assays, and efficacy, mechanistic, and safety assessment in appropriate *in vitro* and *in vivo* models. Abbreviations: MSCs, mesenchymal stem cells; ROS, reactive oxygen species; NAD^+^, nicotinamide adenine dinucleotide; OXPHOS, oxidative phosphorylation; EVs, extracellular vesicles.

As summarized in Figure 3, rejuvenation of senescent MSCs should not rely on correction of a single senescence-associated marker. Instead, it should integrate optimization of culture and manufacturing conditions, restoration of mitochondrial and metabolic homeostasis, and remodeling of epigenetic, proteostatic, and paracrine networks. Importantly, the effectiveness of these interventions should be evaluated using a function-oriented and safety-anchored framework that incorporates identity stability, genomic/epigenomic safety, disease-relevant potency assays assessing proliferation, migration, differentiation, paracrine/EV function, and immunomodulatory activity, as well as efficacy, mechanistic, and safety assessment in appropriate *in vitro* and *in vivo* models.

## 5 Discussion and future perspectives

MSC senescence is a major constraint on the reliable therapeutic potency of cell products because donor-related factors, manufacturing stress, and pathological microenvironments can progressively erode the functions required for efficacy. A product may therefore retain acceptable viability and canonical surface-marker expression while already showing meaningful functional decline. The translational significance of senescence is thus not simply its detection as a laboratory phenotype, but its potential to undermine product consistency and therapeutic reliability (Robb et al., 2022; Yamamoto et al., 2023; Česnik and Švajger, 2024; Robb et al., 2024; Strecanska et al., 2025).

Future work should move beyond uniform release criteria toward source- and indication-specific potency frameworks that connect manufacturing history with disease-relevant functional assays and safety. Rejuvenation strategies should be judged by reproducible restoration of therapeutic activity, rather than by isolated reductions in markers such as SA-β-gal, p16, or ROS. Equally important, any gain in proliferation or secretory activity must be balanced against preservation of cell identity and genomic/epigenomic stability. Establishing such function-based and safety-anchored evidence will be essential for translating MSC rejuvenation into clinically reliable cell products (Sagaradze et al., 2023; Alsultan et al., 2024; Česnik and Švajger, 2024; Tee et al., 2024; Brito and Trentin, 2025; Strecanska et al., 2025).

Recent recommendations associated with the International Society for Cell & Gene Therapy (ISCT) for the peer-reviewed reporting of MSC clinical trials in autoimmune diseases emphasize transparent documentation of trial design, MSC source, manufacturing and handling procedures, product characterization, and potency-related parameters (Davies et al., 2025). Although these recommendations were developed specifically for autoimmune-disease indications, their underlying principles are broadly relevant to MSC-based therapies and highlight the need for reproducible, mechanism-linked, and clinically interpretable potency-assessment frameworks across different MSC sources and therapeutic applications.
